# Supplementary material for: Highly Porous Expanded Graphite: Thermal Shock vs. Programmable Heating
Source: Materials (Basel). 2021 Dec 13;14(24):7687. doi: 10.3390/ma14247687 (PMC8708924; doi:10.3390/ma14247687)
Supplement: Supplementary file 1 [file materials-14-07687-s001.zip › materials-1470412-supplementary.pdf]

materials

Article

Highly Porous Expanded Graphite: Thermal Shock vs. Programmable Heating

Alexander G. Bannov 1,*, Arina V. Ukhina 2, Evgenii A. Maksimovskii 3, Igor Yu. Prosanov 2, Artyom A. Shestakov 1, Nikita I. Lapekin 1, Nikita S. Lazarenko 1, Pavel B. Kurmashov 1 and Maksim V. Popov 1,4

1 Department of Chemistry and Chemical Engineering, Faculty of Mechanical Engineering, Novosibirsk State Technical University, 630092 Novosibirsk, Russia; a.shestakov25@mail.ru (A.A.S.); lapekin21@mail.ru (N.I.L.); 9139278003@mail.ru (N.S.L.); kurmaschov@gmail.com (P.B.K.); popovmaxvik@gmail.com (M.V.P.)
2 Institute of Chemistry of Solid State and Mechanochemistry, Siberian Branch, Russian Academy of Sciences, 630092 Novosibirsk, Russia; auhina181@gmail.com (A.V.U.); prosanov@mail.ru (I.Y.P.)
3 Institute of Inorganic Chemistry, Siberian Branch, Russian Academy of Sciences, 630090 Novosibirsk, Russia; eugene@niic.nsc.ru
4 N.D. Zelinsky Institute of Organic Chemistry, Russian Academy of Sciences, 119991 Moscow, Russia
* Correspondence: bannov.alexander@gmail.com

Figure S1 shows the Raman spectrum of the IG sample. The I(D)/I(G) ratio was 0.07.

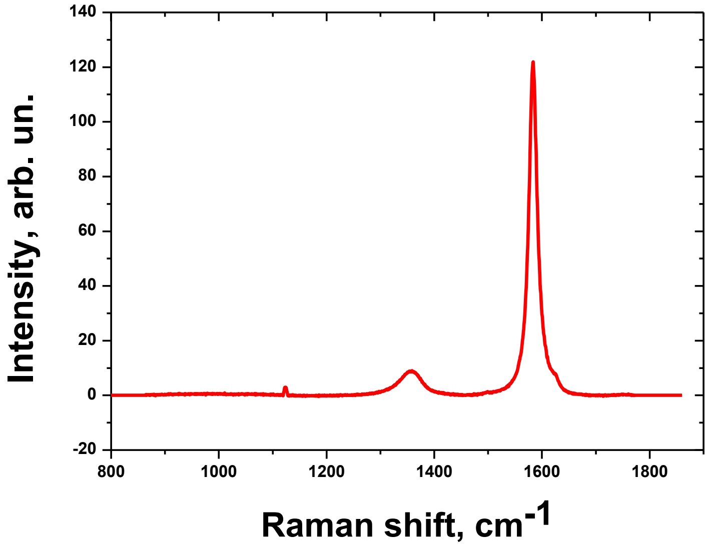

Figure S1. Raman spectrum of the intercalated graphite.

Figure S2 shows the isotherms of adsorption of expanded graphites.

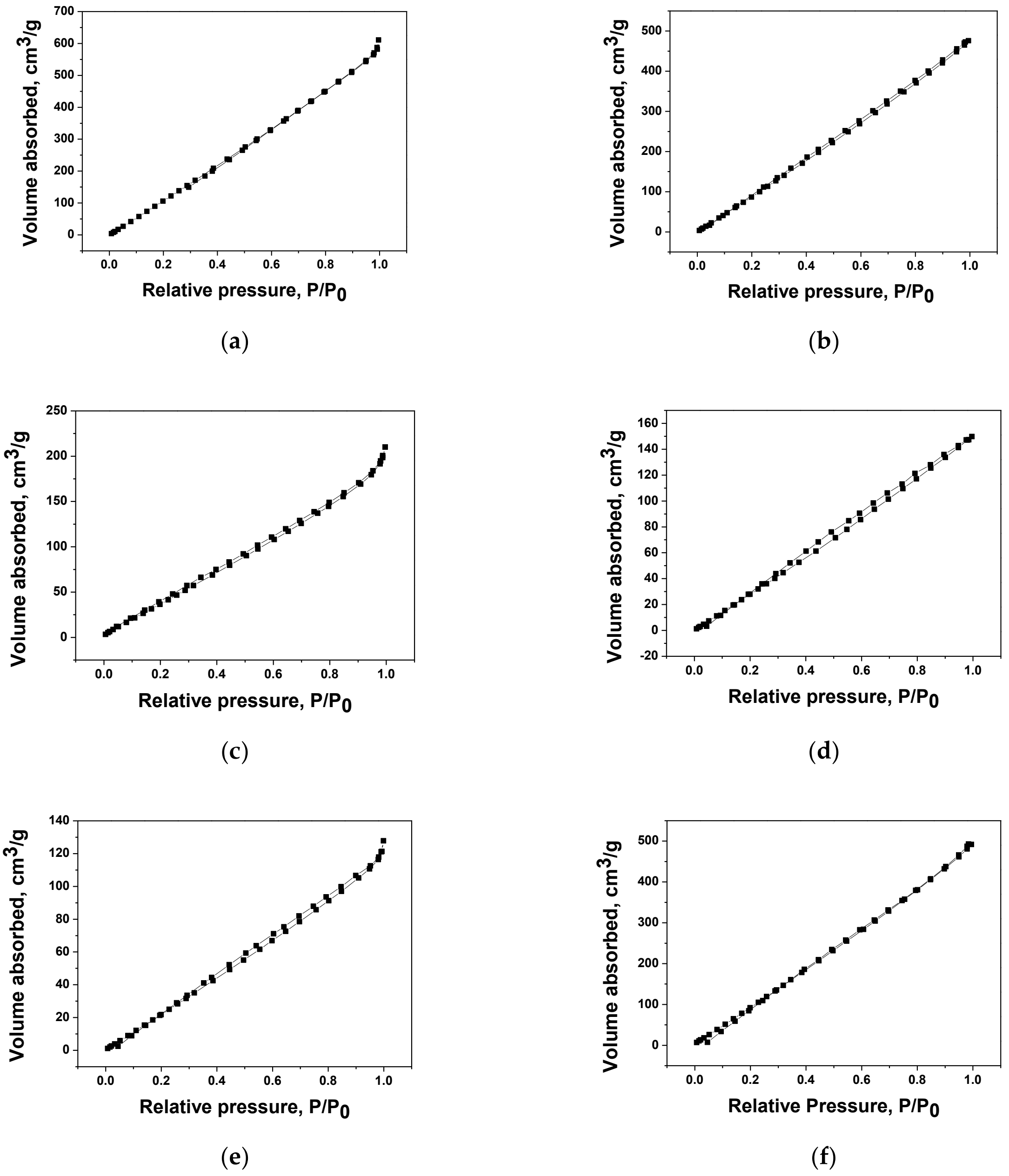

Figure S2. Isotherms of adsorption of expanded graphites: TEG1-400 **(a)**, TEG1-500 **(b)**, TEG1-600 **(c)**, TEG1-700 **(d)**, TEG2-400 **(e),** TEG2-1000 **(f)**.
